# Comparative RNA-seq Analysis in the Unsequenced Axolotl: The Oncogene Burst Highlights Early Gene Expression in the Blastema

**DOI:** 10.1371/journal.pcbi.1002936

**Published:** 2013-03-07

**Authors:** Ron Stewart, Cynthia Alexander Rascón, Shulan Tian, Jeff Nie, Chris Barry, Li-Fang Chu, Hamisha Ardalani, Ryan J. Wagner, Mitchell D. Probasco, Jennifer M. Bolin, Ning Leng, Srikumar Sengupta, Michael Volkmer, Bianca Habermann, Elly M. Tanaka, James A. Thomson, Colin N. Dewey

**Affiliations:** 1 Regenerative Biology, Morgridge Institute for Research, Madison, Wisconsin, United States of America; 2 Technical University Dresden, DFG Center for Regenerative Therapies Dresden, Fetscherstrasse 105 01039 Dresden, Germany; 3 Department of Biomedical Engineering, University of Wisconsin, Madison, Wisconsin, United States of America; 4 Department of Statistics, University of Wisconsin, Madison, Wisconsin, United States of America; 5 Max-Planck-Institute for Biology of Ageing, Cologne, Germany; 6 Department of Cell & Regenerative Biology, School of Medicine and Public Health, University of Wisconsin, Madison, Wisconsin, United States of America; 7 Department of Molecular, Cellular, & Developmental Biology, University of California Santa Barbara, Santa Barbara, California, United States of America; 8 Department of Biostatistics and Medical Informatics, University of Wisconsin, Madison, Wisconsin, United States of America; University of Washington, United States of America

## Abstract

The salamander has the remarkable ability to regenerate its limb after amputation. Cells at the site of amputation form a blastema and then proliferate and differentiate to regrow the limb. To better understand this process, we performed deep RNA sequencing of the blastema over a time course in the axolotl, a species whose genome has not been sequenced. Using a novel comparative approach to analyzing RNA-seq data, we characterized the transcriptional dynamics of the regenerating axolotl limb with respect to the human gene set. This approach involved de novo assembly of axolotl transcripts, RNA-seq transcript quantification without a reference genome, and transformation of abundances from axolotl contigs to human genes. We found a prominent burst in oncogene expression during the first day and blastemal/limb bud genes peaking at 7 to 14 days. In addition, we found that limb patterning genes, SALL genes, and genes involved in angiogenesis, wound healing, defense/immunity, and bone development are enriched during blastema formation and development. Finally, we identified a category of genes with no prior literature support for limb regeneration that are candidates for further evaluation based on their expression pattern during the regenerative process.

## Author Summary

Salamanders such as the axolotl can fully regenerate a limb upon amputation, making them the vertebrate champions of regeneration. On the other hand, humans and other mammals possess a very limited ability to regenerate limb structures. Learning about the genes, gene networks, and pathways activated in the salamander during limb regeneration will provide cues to improving the regenerative response in mammals. Elucidating these genes, networks, and pathways is difficult, however, because the axolotl does not yet have its genome sequenced and because it has diverged evolutionarily from species with a sequenced genome. Here, we produce a set of gene transcripts via RNA sequencing (RNA-seq) for the axolotl and provide information on the nature of the genes activated during regeneration. To determine the identity of these axolotl genes, we use comparative transcriptomics techniques to match the axolotl transcript data to that of the well-annotated human gene set. Supporting previous studies, we find upregulation of many genes previously found to be involved in limb development and regeneration. In addition, we find a burst of cancer-related genes during the first phase of regeneration and identify a set of genes previously not associated with the regeneration process.

## Introduction

The salamander's capability to regenerate various body parts, including limbs, has been the focus of study for almost 200 years [1]. Regeneration of the limb of the axolotl (*Ambystoma mexicanum*), in particular, has been widely studied, both in classical studies, and more recently in studies using modern molecular tools [2]–[10].

Recent publications have incorporated microarray, sequencing, and mass spectrometric technology into the study of limb regeneration. For example, a study focusing on the nerve dependence of limb generation utilized microarrays and 454 sequencing to identify genes expressed in the blastema with and without the presence of nerves [3]. A more recent study utilized a microarray with 20,000 probe sets to identify genes expressed during the regenerative process [11]. However, there are limitations to this approach, as microarrays can only identify the expression of genes for which probes are designed, and the shallow depth of 454 sequencing in the first study limits the ability to detect low abundance transcripts. Another study in *Xenopus* identified several genes likely to be important to hind limb regeneration [12], and a proteomic analysis of the blastema has also been published [2]. More recently, a study employed microarrays to identify a list of genes specific to the regenerating epithelium [13]. In addition to the above axolotl studies, Expressed Sequence Tag (EST) and other sequencing efforts have made inroads in defining the transcriptome of the newt [14]–[16].

In our study, we examine the axolotl transcriptome using RNA-seq technology, which can provide accurate expression level estimates for genes across a wide range of abundances [17]. To the best of our knowledge, a deep sequencing of the developing axolotl blastema using RNA-seq has not yet been reported. Despite its advantages, RNA-seq data analysis for the axolotl is challenging as the species' genome has not yet been sequenced, probably due to its size (∼30 GB) [18], and its transcriptome is largely uncharacterized. To address these challenges, we have developed a novel computational approach to the analysis of axolotl RNA-seq data that allows for characterization of axolotl transcriptional dynamics in terms of the human gene set. Our approach uses statistical tools for transcript quantification when no reference genome is available [19] and comparative transcriptomic analysis with the human transcript set.

Given the critical role played by the blastema in the regenerative process [20], we used RNA-seq and our novel comparative analysis approach to uncover the sets of genes regulated in the blastema over the first 28 days after amputation. We established a website that contains all of the RNA-seq read and assembly information from this work and other relevant “omic” information on the axolotl (www.axolomics.org). This paper provides information on genes and gene products likely important for the maintenance, growth and proliferation of stem cells, and for the regulation of growth and tumor formation. It supplies critical resources for a deeper understanding of the blastema's contribution to limb regeneration and lays the groundwork for important advances in the field of regenerative biology.

## Results

### Sequencing of blastemal RNA

**Figure 1:**
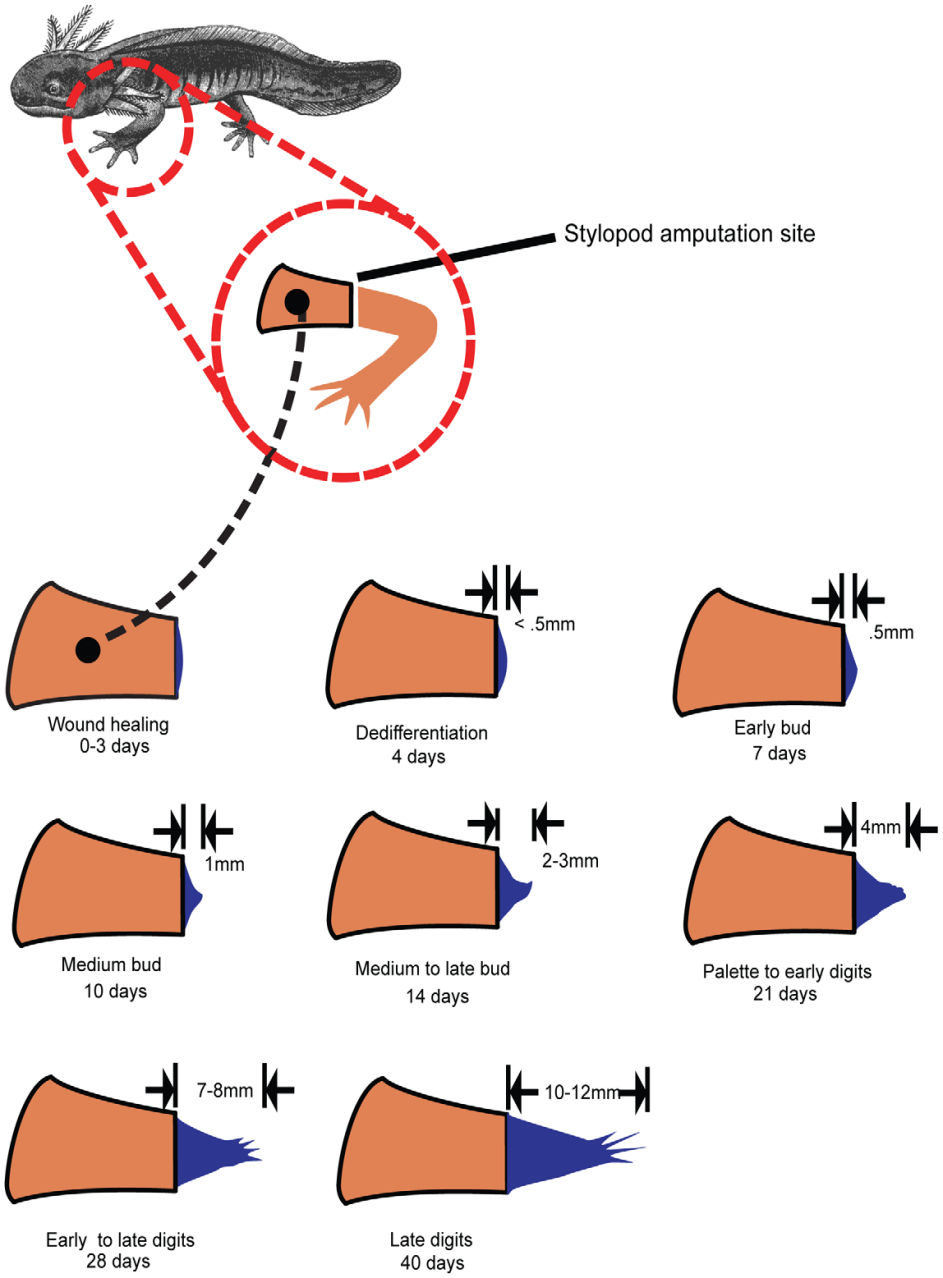
Diagram of the blastemal time course experiment. RNA was harvested from tissue samples and subjected to deep sequencing on the Illumina GA II platform (see also Figure S1).

We amputated juvenile (4.5–10 cm) axolotl right forelimbs at the mid-stylopod level and harvested tissue at 0 hours, 3 hours, 6 hours, 12 hours, 1 day, 3 days, 5 days, 7 days, 10 days, 14 days, 21 days, and 28 days (Figure 1). We focused on forelimbs because gene expression patterns can differ between forelimb and hindlimb during regeneration [21] and used only right limbs because left-right expression asymmetries may also exist. Tissue from three animals at each time point were pooled to reduce the impact of individual variation. We then prepared mRNA and performed sequencing using the Illumina GAII platform (see Materials and Methods).

### Quantification of transcript abundances with respect to the human gene set

Full details of our comparative RNA-seq analysis approach are provided in Materials and Methods. Briefly, RNA-seq reads were first mapped to an axolotl mRNA contig set containing 113,925 contigs. These contigs were then matched to human transcripts to allow for differential expression and functional analyses using the human gene set.

Read counts and measures of Transcripts Per Million (TPM) were calculated for each human gene matched by at least one contig [22]. The numbers of reads sequenced and mapped to axolotl contigs and human genes for each sample are provided in Table S1. The validity of this approach can be seen through analogy with standard methods for gene-level RNA-seq analyses, which typically sum the counts of reads mapping to each exon or isoform of a gene to arrive at a gene-level count. In our case, the axolotl contigs are simply treated as “isoforms” of the human genes to which they are matched.

Other quality axolotl transcriptome assemblies exist. A recent study producing axolotl ESTs resulted in 15,384 contigs, but these contigs are derived from brain sampling and thus may not give a good representation of genes active in limb regeneration [23]. Sal-Site (www.ambystoma.org) also provides a web-searchable assembly of 17,000 contigs that map to human genes. We chose to use our assembly because many of the tissues used to construct the assembly are derived from the limb or blastema.

Our samples have, on average, 10,242 human genes with at least one read associated with them and, on average, samples have 9,285 human genes with a TPM>1 (Figure S1). Analyzing all samples, we identified 11,927 genes with reads (47% of the 25,484 protein-coding gene symbols in the human gene set). Given that approximately 60–70% of genes in the genome are expected to be expressed in any particular cell type, the 47% detected in the present study likely represents a substantial percentage of the genes actually expressed in the axolotl blastema [24].

We further characterized the completeness of our axolotl transcriptome through an analysis of the high-quality RNA-seq reads without an alignment to an axolotl contig. A fraction of these unalignable reads are true axolotl sequences that could not be mapped due to an incomplete assembly while the remainder of these reads are probably RNA-seq artifacts, such as adapter sequences. To estimate these fractions we ran BLASTX on both high-quality alignable (AL) and unalignable (UN) RNA-seq reads against the human protein set. We found that 17% of AL reads had a significant BLASTX hit, while only 4% of UN reads had such a hit. We thus estimate that the fraction of unalignable reads that are truly from axolotl is 23% = 4%/17%. Given that 49% of all reads were unalignable, we estimate that 11% = 49%×23% of all reads were from axolotl but were unalignable either due to an incomplete assembly or sequencing error. Examining the sets of human proteins hit by the AL and UN reads, we found that 73% of the human proteins hit by either the AL or UN reads are hit by the AL reads. Thus we estimate that our axolotl assembly represents 73% of the transcriptome of our sampled tissues. A key assumption used by these analyses is that the transcripts in our axolotl assembly are not biased with respect to their sequence similarity to the human protein set. Although this assumption is not likely to be true across the board, we have no reason to believe that our assembly would be heavily biased in this respect. For additional details on the logic behind these calculations, see Materials and Methods.

Our approach is dependent on comparative techniques between axolotl and human transcriptomes, and thus it excludes salamander clade-specific genes. To address this, we mapped contigs to salamander genes available in NCBI (as of 5-23-2011), and recovered 677 salamander genes through this analysis. 614 of these 677 genes have reads associated with them. However, 88% of the contigs that mapped to a salamander gene were also mapped to a human gene. We also identify ∼80,000 contigs with BLAST e values > = 10^−5^ (to both salamander and human gene sets), which are generally shorter (median length = 480) than contigs with significant BLAST hits (median length = 602). While these contigs lack ties to human and salamander annotations, the expression patterns of many of them indicate that they likely are involved in the regenerative response. (Contig expression data is available on www.axolomics.org.)

### Data quality

**Figure 2:**
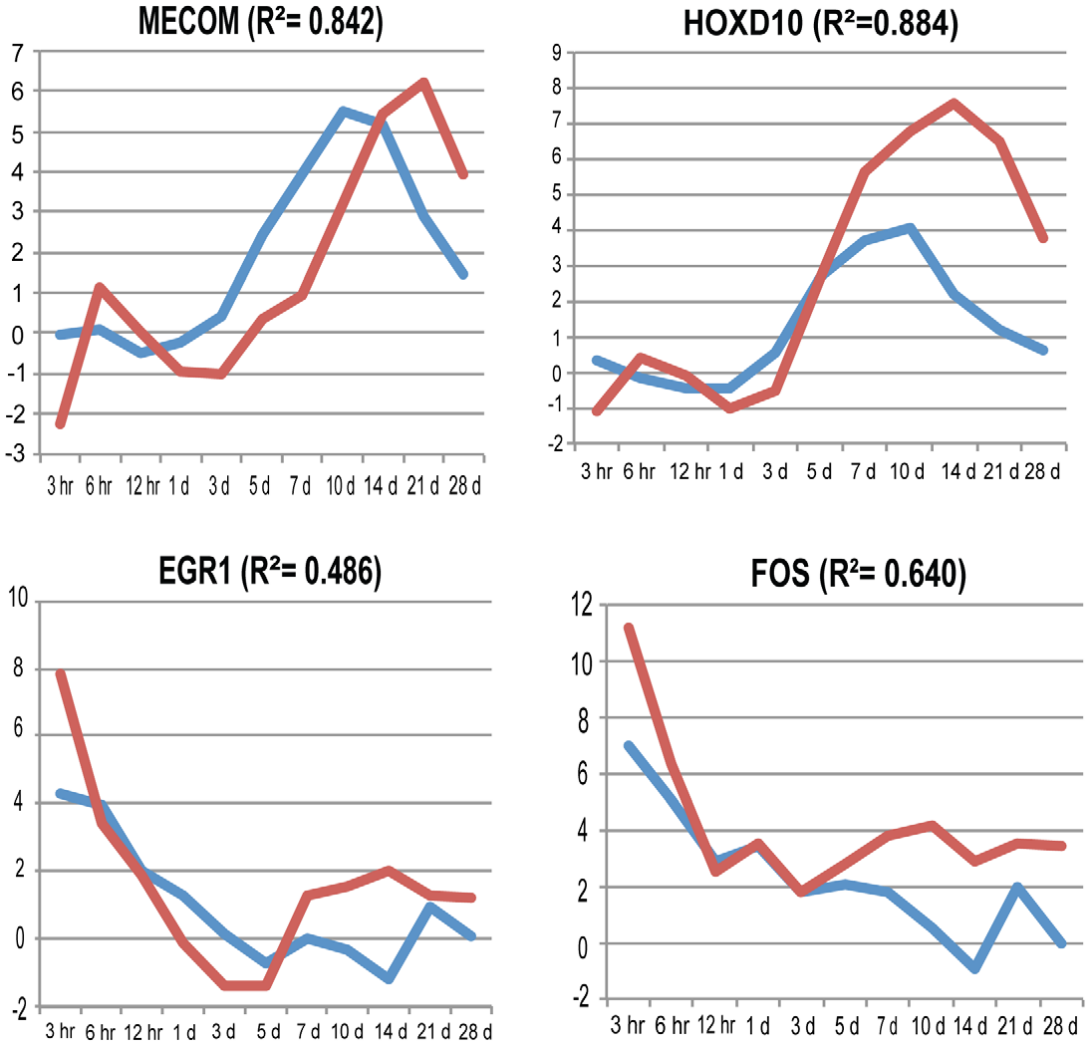
Expression patterns of RNA-seq and qPCR along the time course. Plots of the log_2_ ratios of TPMs (transcripts per million) of each time point relative to the time zero control for RNA-seq data (blue), and the log_2_ ratios of the qPCR expression measure (red) relative to the zero hour control. The R^2^ Pearson correlation across the time course is shown for each gene as well. The expression patterns are similar between the RNA-seq and qPCR data (average Pearson correlation for the four genes shown = 0.839, average Pearson correlation for all 19 genes = 0.741). See Figure S2 for the 15 additional genes not shown in Figure 2, and Table S2 for all Log_2_ ratios of RNA-seq and qPCR data. See Materials and Methods for an explanation of determining the ratios and for the Pearson calculation.

We performed differential expression (DE) analysis through the time course comparing each time point after the zero hour control to the zero hour control using EdgeR (see Materials and Methods for details) [25]. For all downstream analyses, we consider only those genes with a false discovery rate (FDR)<0.05. To assess RNA-seq data quality, we chose 19 genes from the DE set, and performed real-time quantitative PCR (qPCR) on biological replicates in triplicate (and triplicate technical qPCR replicates) as an orthogonal analysis. We found that the expression pattern (relative to the zero hour control) matches closely in all 19 genes (average Pearson coefficient for all 19 genes = 0.741) (see Figure 2, Figure S2 and Table S2). Four of these genes are shown in Figure 2. These data indicate that our RNA-seq data accurately represent expression. The log2 ratios relative to the zero hour control for all 19 genes for both the RNA-seq data and qPCR data is shown in Table S2.

### Comparison of the comparative RNA-seq approach to a simpler contig-level approach

Our comparative approach to DE analysis of RNA-seq data may be contrasted with a simpler approach that performs the analysis directly at the level of individual contigs. In this contig-level approach, read counts are estimated for individual contigs in each sample and used to predict contigs that are DE (see Materials and Methods). Contigs predicted to be DE may then be functionally analyzed through annotations obtained by BLAST hits to a well-characterized gene set, such as that of the human.

We implemented the contig-level DE approach and found that it predicted 3,671 contigs as DE at any time point. These 3,671 DE contigs mapped to 1,040 human genes, a significantly smaller set (p<2.2e-16, Fisher's exact test) than the 1,656 genes predicted as DE by the comparative approach. The smaller number of DE genes predicted by the contig-level approach is probably due to the fact that read counts had a significantly higher variance (p<0.002, Wilcoxon signed rank test) at the contig-level (median edgeR common dispersion = 0.11) than at the human gene level (median edgeR common dispersion = 0.05). Read count estimates have higher variance at the contig level because contigs are typically shorter than full-length transcripts and often have high sequence similarity to other contigs that may represent alternative isoforms of the same gene. Thus, the comparative approach gains more statistical power for DE tests through its use of less variable counts at the human gene level.

Of the 19 genes validated by qPCR that were called DE by the comparative gene-level approach, only 13 were called as DE by the contig-level method, and of the 19 contigs used for qPCR primer design, only 10 were called as DE. Given that the qPCR data support that these 19 genes and contigs were all DE across the time course, these results indicate that the comparative approach has superior statistical power. Examining the two DE gene sets, 709 genes are called as DE by both approaches. Of the 331 DE genes uniquely called by the contig-level method, two were identified as limb development genes (based on the 101 limb genes in Figure S3), whereas the 947 DE genes uniquely called by the comparative method included 19 of these 101 limb development genes. This result also indicates that the comparative approach had more power on our data, although the difference between the numbers of limb development genes uniquely called by the two methods is not statistically significant (p = 0.13, Fisher's exact test).

Interestingly, a large fraction (53%) of the DE contigs did not have significant BLAST hits with the human gene set. These contigs were somewhat shorter (640 bp median length) than DE contigs with a significant BLAST hit (727 bp median length, p<2.2e-16, Wilcoxon rank sum test). More revealingly, through an analysis of the lengths of the longest open reading frames (ORFs) within the DE contigs, we found that the DE contigs without a significant BLAST hit generally had a much smaller fraction of their length contained within their longest ORF (Figure S4). In addition, the distribution of the fractional lengths of the ORFs in shuffled contig sequences was relatively similar to that in the DE contigs without significant BLAST hits. These results suggest that these contigs are largely non-coding and may be derived from non-coding genes or the UTRs of protein-coding genes. Thus, the DE results obtained from our comparative method are likely capturing the vast majority of the protein-coding genes involved in axolotl limb regeneration.

### Patterns of gene expression along the juvenile blastemal time course: early oncogenes, later limb/blastemal genes

**Figure 3:**
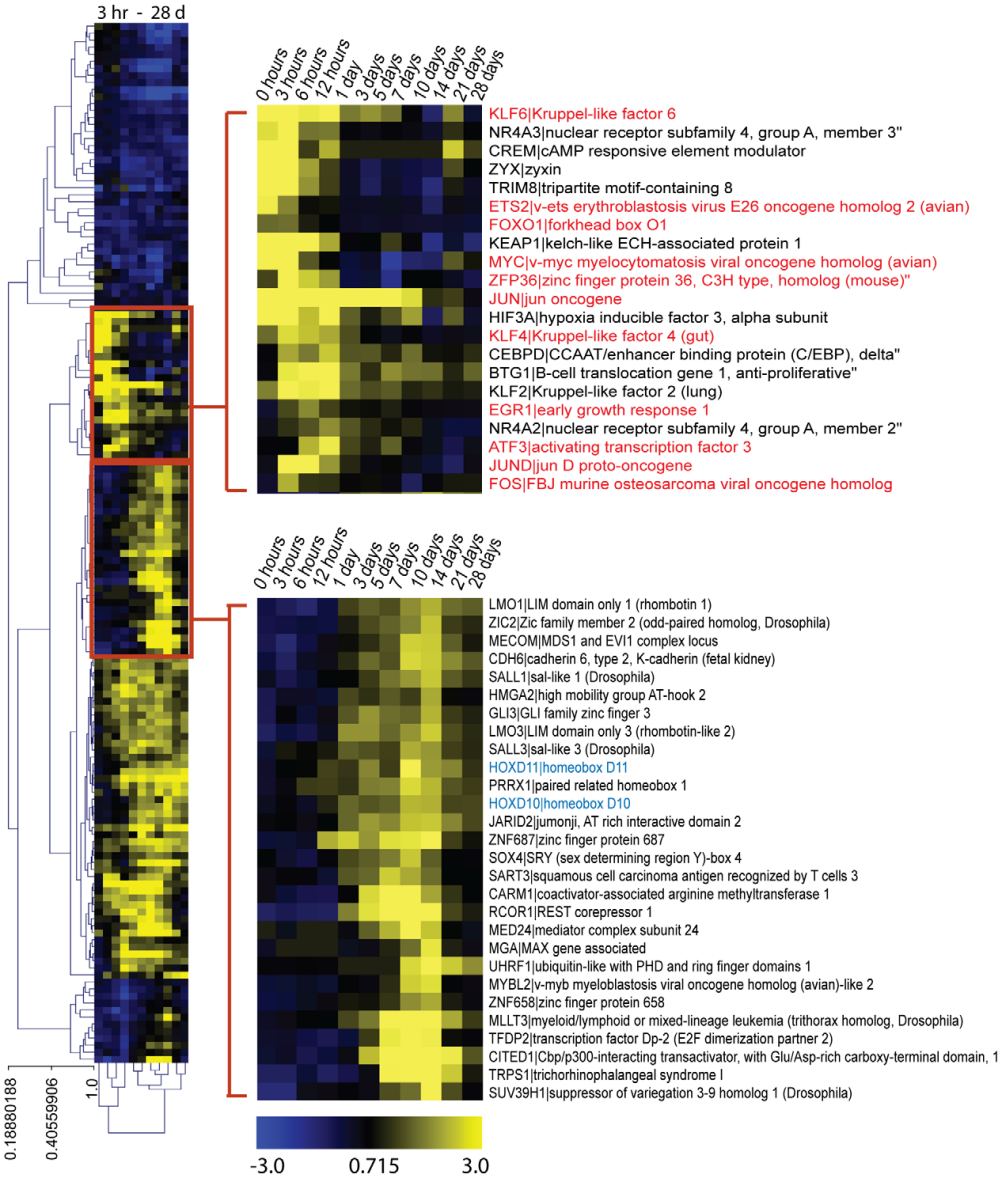
Two-way clustering of all differentially expressed (DE, FDR<0.05) transcription factors (TFs). Differential expression is relative to the zero hour control. Upper right: an early upregulated cluster of TFs dominated by oncogenes (ATF3, KLF4, KLF2, JUN3, EGR1, NR4A2, and FOS are all oncogenes). Oncogenes are highlighted with red text. Lower right: a cluster of TFs upregulated later in the time course including genes involved in limb development such as HOXD10 and HOXD11 (highlighted in blue text). Clustering and viewing was done with Mev 4.8.1 The distance measure used was the Pearson uncentered correlation with average linkage.

**Figure 4:**
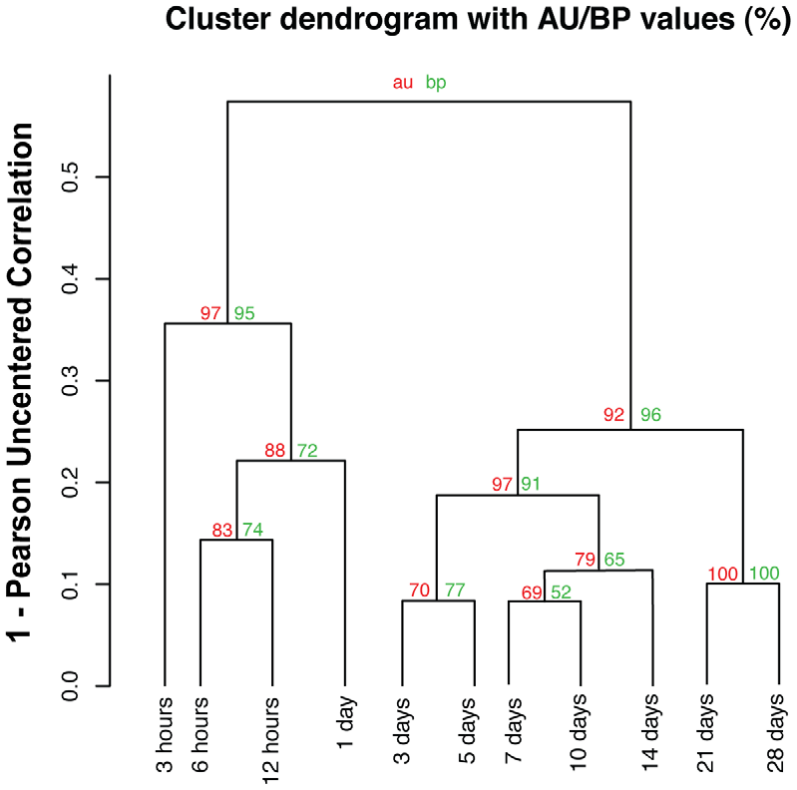
Bootstrapping of the sample clusters from **Figure 3**. To assess the uncertainty in hierarchical cluster analysis over samples, bootstrap resampling (10,000 iterations) was applied via the R package Pvclust [26]. The uncentered Pearson correlation is used as the distance metric with average linkage. The numbers above each edge show the probability of nodes below that edge occurring as a cluster in resampled trees, via ordinary bootstrap resampling (BP, green) or multiscale bootstrap resampling (AU, red). See Materials and Methods for details on bootstrapping.

We evaluated the entire list of DE genes and a subset of that list containing only transcription factors (TFs). Global DE statistics are available in . (And lists of all significantly differentially expressed genes and the TF list are available at www.axolomics.org.) We performed two-dimensional clustering of all DE TFs along the time course (Figure 3), and found that oncogenes dominate during the first day, while TFs associated with limb development or regeneration, such as HOXD10 and HOXD11, peak at days 10 to 14 (Figure 3). We assessed the confidence of our sample clustering by performing cluster bootstrapping analysis (10,000 iterations) with the R package PVClust. PVClust gives both a bootstrap proportion (BP) measure as well as an approximately unbiased (AU) measure (see Materials and Methods) [26]. This bootstrap analysis provides statistical support for the existence of an early cluster (day 1 and earlier samples) and a later cluster (day 3 and later samples) (Figure 4). A heat map showing the pairwise Pearson coefficient correlations between each of the time points is shown in Figure S6 (and all of the correlation values can be found in Table 3).

**Figure 5:**
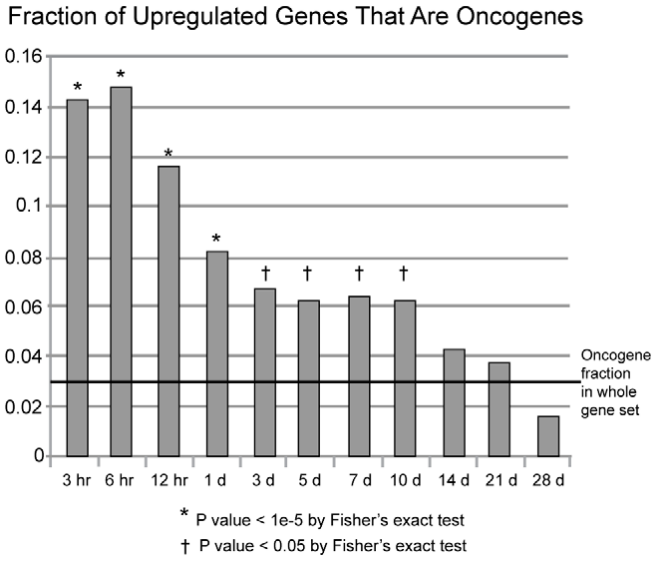
Enrichment of oncogenes during the early time points. All upregulated DE genes at each time point (when compared to the zero hour control) were interrogated for being oncogenes by their presence in the Memorial Sloan Kettering Cancer Center Oncogene set (http://cbio.mskcc.org/CancerGenes/Select.action) and then performing a Fisher's exact test to assess significance. The enrichment of oncogenes during the first day is highly statistically significant (P-value<10^−5^). Late in the time course, oncogenes are not significantly enriched in the upregulated DE gene sets.

The cluster of TFs upregulated during the first day include many known oncogenes (e.g. *ATF3*, *EGR1*, *ETS2*, *FOS*, *FOXO1*, *JUN*, *JUND*, *KLF4*, *KLF6*, *MYC*, *ZFP36*) (Figure 3, upper right cluster) [27], [28]. The observation of a burst of oncogenes early in the formation of the blastema is novel. We evaluated the statistical significance of oncogene upregulation throughout the entire time course by determining the number of upregulated oncogenes (based on the list of oncogenes available from the Memorial Sloan Kettering Cancer Center at http://cbio.mskcc.org/CancerGenes/Select.action) and by performing a Fisher's exact test to measure significance (Figure 5). Note that oncogenes are very significantly enriched in the upregulated DE set (p<10^−5^) during the first day, and continue to show some enrichment (p<0.05) through day 10. Later in the time course, oncogenes are not significantly enriched in the upregulated DE gene sets. We also find significant enrichment of oncogenes in upregulated DE gene sets of the time points of the first day as a group (0 hr, 3 hr, 6 hr, 12 hr, 1 d) compared to later time points as a group (3 d, 5 d, 7 d, 10 d, 14 d, 21 d, 28 d) (p = 0.018).

In addition to TFs, we evaluate all DE genes. Among the most significantly upregulated genes during the first day are matrix metalloproteinases (*MMP1*, *MMP2*, *MMP3*, *MMP8*, *MMP9*, *MMP10*, *MMP12*, *MMP13*, *MMP12*, *MMP19*), many of which were observed previously [3], [29]. (See axolomics.org for lists of upregulated and downregulated genes and the subset of TFs. Files are provided for up and down regulation at each time point, and consolidated files showing all genes upregulated or downregulated at any point along the time course (prefix = wholeTC)). Another category of genes expressed during this time period are the dual specificity phosphatases, DUSP1, DUSP5, and DUSP7, which are involved in MAP/ERK signaling and can play roles in development or cancer [30].

Later in the time course (between 3 d–14 d) there is a prominent cluster of genes (Figure 3, lower right cluster), many of which are involved in limb development or patterning. Expression of oncogenes early in the time course and limb genes later corresponds to the two well-recognized phases of limb regeneration, the early “preparatory” and later “redevelopment” phases respectively [31]. Upon examination of the DE lists, significantly upregulated genes include HMG group genes (*HMGA1*, *SOX11*, *SOX4*, *HMGA2*) and genes known to be expressed in blastemas or limb buds, or to be involved in limb development and patterning (*PRRX1*, *HOXD10*, *PRDM1*, *SALL1*, *TBX18*, *SALL4*, *HOXD11*, *GLI3*, *SALL3*, *TGFB1*, *TNC*) [31]–[40]. Also upregulated during this time include genes upregulated in embryonic or adult stem cells (*JARID2*, *SALL1*, *ZIC2*, *HMGA2*, *SALL4*) [41]–[45]; SHH, a gene crucial to limb regeneration, and SUFU, which regulates SHH [46]; tumor suppressor genes (*APC*, *SMAD4*) [47]; and a component of the polycomb complex (*EED*) [48]. It is also noteworthy that many limb genes peak at day 10 or 14 (Figure S3).

At the end of the time course (21 d–28 d), expression of the HMG genes, limb genes, tumor suppressor, polycomb, and stem cell genes decreases so that, of the 24 genes listed above for the 3 d–14 d time points, only one gene (*SALL1*) remains upregulated by days 21–28. During the last two time points, upregulated genes include keratins, collagens, and genes associated with collagen or cartilage formation (EPYC, MATN4) (genecards.org).

**Figure 6:**
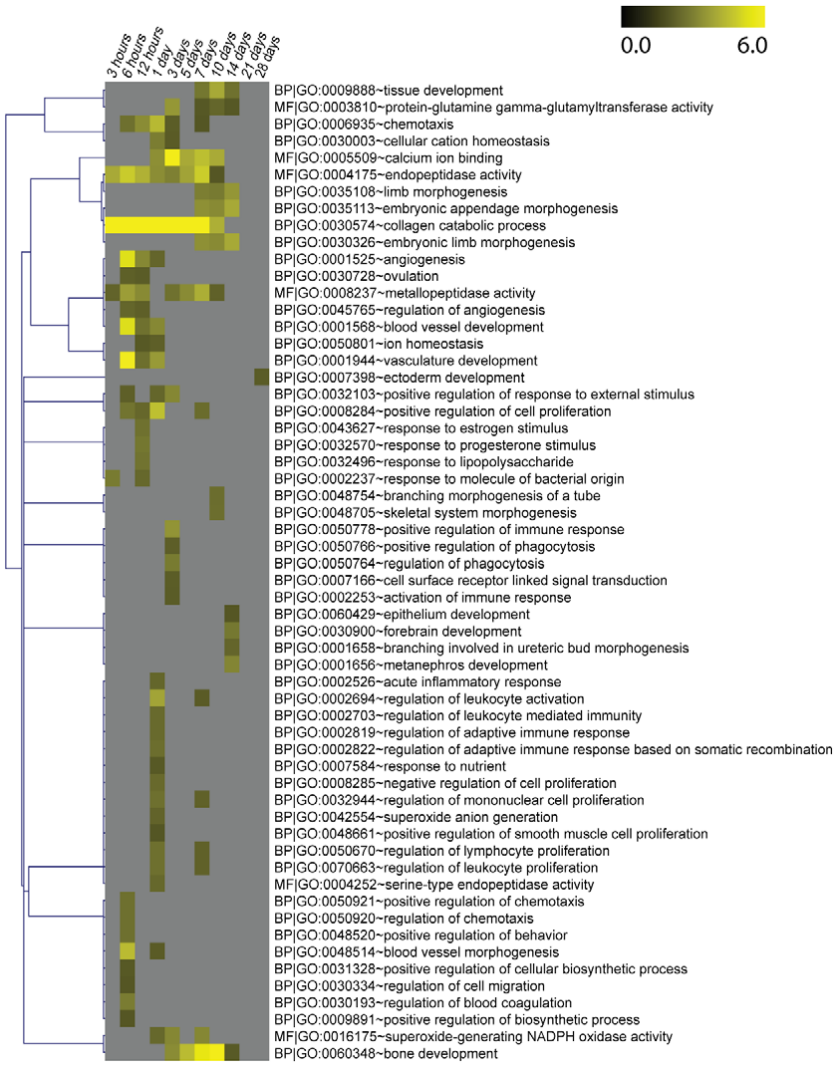
Gene ontology (GO) analysis of the time course. GO enrichment of molecular function (MF) and biological process (BP) level 5 GO terms during the time course for all DE genes (relative to the zero hour control). The value displayed is the −log_10_(GO term FDR). GO terms with an FDR<0.01 are shown. The GO terms were clustered by their enrichment patterns using the Pearson correlation as the distance measure with average linkage. During the first day enriched GO terms include GO terms representing immune response, chemotaxis, regulation of leukocytes, blood vessel development, and angiogenesis. In the middle of the time course (3 d–14 d), GO terms for tissue development, limb morphogenesis, bone development, and forebrain development are enriched. An ectoderm development GO term is enriched at 28 days. (Detailed GO enrichment information for upregulated axolotl genes is available at www.axolomics.org). (See also Table S4 for the −log_10_(GO term FDR) values.)

We used DAVID (Database for Annotation, Visualization and Integrated Discovery) to perform gene ontology (GO) analysis for biological process (BP) and molecular function (MF) GO terms at level 5 (see Materials and Methods) on all DE genes [49]. The results are shown in the heat map in Figure 6. The GO data for the figure is available in Table S4. During the first day enriched GO terms include GO terms representing immune response, chemotaxis, regulation of leukocytes, blood vessel development, and angiogenesis. In the middle of the time course (3 d–14 d), GO terms for tissue development, limb morphogenesis, bone development, and forebrain development are enriched. An ectoderm development GO term is enriched at 28 days. (Detailed GO enrichment information for upregulated axolotl genes is available at www.axolomics.org).

### Relationship of blastema cells and stem cells

**Figure 7:**
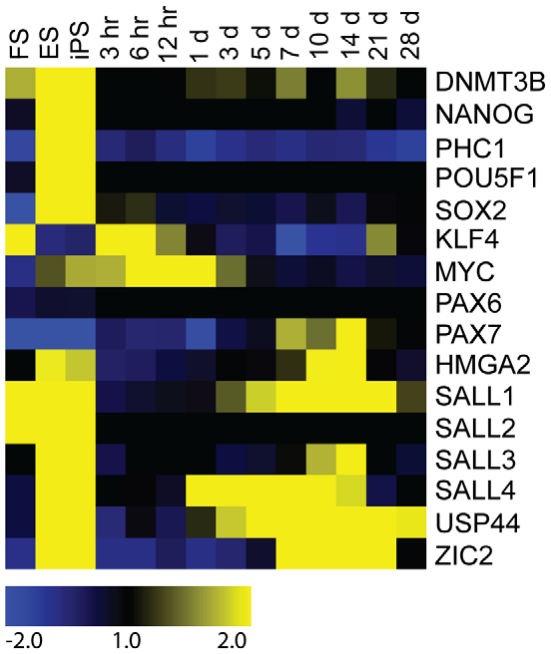
Expression of embryonic and adult stem cell genes in ES, iPS, FS, and blastemas. All samples are ratioed to the time zero axolotl juvenile time course control. While ES and iPS cells show upregulation of a variety of stem cell genes including the key ES transcription factors *POU5F1* and *NANOG*, the axolotl blastemas do not show upregulation of *POU5F1*, *SOX2*, and *NANOG*, but do show upregulation of adult stem cell markers (such as *SALL4* and *HMGA2*). Note the burst of oncogenes (*KLF4*, *MYC*) early in the time course and the upregulation of adult stem cell genes later in the time course.

We performed RNA-seq on human embryonic stem (ES) cells, human induced pluripotent (iPS) stem cells, and human foreskin (FS) cells, and compared the expression of ES and stem cell genes in these cells and blastemas (see axolomics.org for reads, alignments and expression measures of ES, FS, and iPS cells). Whereas blastemal cells do express some embryonic stem (ES) cell genes, they are distinct from pluripotent cells and bear more in common with adult stem cells. We found upregulation of induced pluripotent stem (iPS) cell reprogramming factors *KLF4* and c-MYC in blastemas in agreement with prior work [50], [51]. SALL4, which is thought to be involved in the maintenance of embryonic and/or adult stem cells [52], [53], is significantly upregulated during the time course. However, the key embryonic stem cell markers *POU5F1* (OCT4), SOX2, and *NANOG* are not highly upregulated in blastemas (Figure 7). PAX7 is upregulated and HMGA2 is significantly upregulated in the blastemas (Figure 7), and both are markers of adult stem cells [42], [54].

## Discussion

Evaluation of gene expression patterns in the blastema time course reveals a prominent burst of oncogene expression during the first day of blastema formation. In addition, our blastema-enriched gene set confirms many known findings from the limb development and limb regeneration literature including genes known to be expressed in the blastema or limb bud. This fact, when combined with qPCR validation of biological replicates, suggest that the RNA-seq data is robust and that the many novel blastemal-enriched genes we discover are strong candidate genes crucial for regeneration. Some of these novel genes include SALL genes, putative patterning and limb development genes, oncogenes, and HMG genes. These genes play roles in the establishment or maintenance of the stem cell state, chromatin accessibility, proximal/distal patterning, and cell cycle control. Novel gene categories are discussed below.

### SALL genes

**Figure 8:**
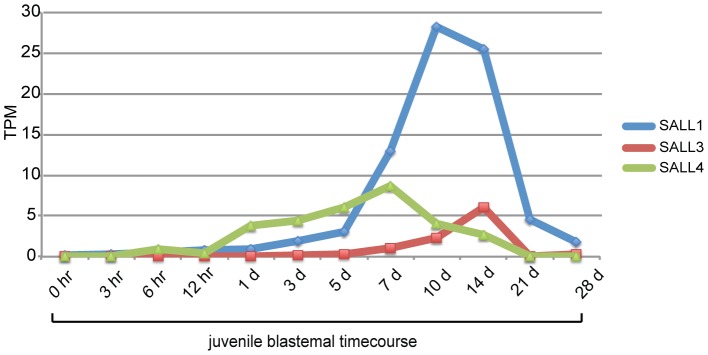
Expression of *SALL1*, *SALL3*, and *SALL4* in juvenile blastemas. *SALL4* is upregulated earlier than *SALL1* or *SALL3*. “TPM” is the Transcripts Per Million expression measure.

The SALL genes (*SALL4*, *SALL3*, *SALL1*) are among the most highly upregulated TFs. *SALL4* has been shown to play a role in *Xenopus* limb development, and thus is a strong candidate for being involved in the regenerative process [40]. The concordance of axolotl blastemal expression patterns of *SALL1*, *SALL3*, and *SALL4* with those in *Xenopus* supports the suggestion that *SALL4* plays a role in initiating blastema cell formation while *SALL1* and *SALL3* are involved in patterning events [37], [40] (Figure 8). The continued expression of *SALL4* is consistent with data indicating that *SALL4* is also involved in limb patterning [55]. *SALL4* is one of the few TFs thought to play a role in maintenance of the ES state and in the putative maintenance of adult stem cells [56], [57]. It is possible that *SALL4* is important for establishment or maintenance of the multiple adult stem cell types thought to be present within the blastema. In support of its regeneration-specific role, *SALL4* is upregulated during *Xenopus* limb regeneration, but a wound without amputation in *Xenopus* does not result in *SALL4* upregulation [40]. Similarly, *SALL4* is upregulated in innervated axolotl blastemas when compared to a non-regenerative flank wound [11].

### Proximal/Distal patterning and limb development genes

Important homeobox (HOX) genes that are markers of, and perhaps specifiers of, proximal/distal pattern (*HOXD10*, *HOXD11*) are upregulated during the juvenile time course. Many other genes (see Figure 3) share expression patterns similar to *HOXD10/11*. Some of these genes have known roles in limb development (such as GLI3), however most have no known role in pattern formation or limb development. The genes in this expression cluster thus become candidate genes for playing a role in pattern formation or other aspects of limb redevelopment during limb regeneration. All nine genes chosen from this cluster for further validation by qPCR (CDH6, GLI3, HMGA2, HOXD10, LMO1, MECOM, PRRX1, SALL3 and SALL1) have corroborating qPCR evidence for this expression pattern (Figure 2 and Figure S2).

### Oncogenes and tumor suppressors

Analysis of genes expressed in the blastema over the time course indicates that oncogenes are activated early in the process and then downregulated. Oncogenes play multiple roles during development and may be crucial for opening chromatin to reactivate developmental programs. During the early time periods of blastema formation, both dedifferentiation and proliferation are thought to occur [7], and oncogenes may be playing roles in both of these processes. RAS and JUN family oncogenes are upregulated during newt lens regeneration [16].

Tumor suppressors are also upregulated in the juvenile blastema. It is possible that tumor suppressors and oncogenes maintain a critical balance in the blastema. *HMGA2*, which is highly upregulated in the blastema, inactivates the tumor suppressor *ARF* in neural stem cells [42]. *ARF* inactivation has been shown to be important for activating regenerative muscle cells in the mouse [58]. The interplay between oncogenes and tumor suppressors may be crucial for the maintenance of multiple adult stem cell types within the blastema. The fact that salamanders are resistant to carcinogens [9], [59] may be a result of the ability to control oncogenes during the regenerative response.

### HMG genes and chromatin modifiers

HMGA1 and HMGA2 are highly over-represented in the blastema. HMG genes play a role in adult stem cells and in opening chromatin, preventing differentiation, and encouraging self-renewal of stem cells [42], [60]. *HMGA2* has been identified as a promoter of self-renewal and is more highly expressed in young adult stem cells than older adult stem cells [42]. Our observed upregulation of *HMGA2* in the blastema might be an indicator of the presence of actively renewing adult stem cells in the blastema. The axolotl blastema is likely composed of a variety of adult stem cells each with its own limited differentiation capacity [4]. Likewise, the zebrafish jaw blastema appears composed of distinct progenitors for muscle and skeleton [61]. In the present study, no attempt is made to separate out the various progenitor cell types within the blastema. The upregulated genes identified here provide a springboard for the development of markers and affinity reagents for dissecting out the various cell types within the blastema. For instance, further studies using in situ hybridization for highly upregulated factors could potentially identify progenitor cell-specific transcripts based on position within the developing blastema, time of expression, and/or co-staining with mature cell-specific markers (e.g. muscle markers, cartilage markers, nerve markers), which would be expressed later in the time course or during limb regeneration. Finally, the upregulation of *HMGA2*, with its association with young stem cells, is consistent with the suggestion that the blastema is a “young” tissue where it is recapitulating developmental patterns of gene expression resulting in a possibly rejuvenated limb after regeneration [62]. Verification of this possibility will require investigation of telomere length and other markers of aging before and after regeneration.

Reprogramming differentiated cells back to the appropriate adult stem cell state likely involves chromatin remodeling and suppression of parental gene expression programs via the polycomb group (PCG) complex. Blastema-enriched genes include several involved in chromatin remodeling, the PCG complex, gene silencing, and DNA methylation (*UHRF1*, *EED*, *SETDB1*) [63]–[65].

### Other genes

In addition to limb development or patterning genes, HMG genes, and oncogenes, we found several other interesting genes upregulated over the time course including *MYF5 and FOXO1* (likely involved in regulation of myogenesis), *RUNX1*, *SPI1* (PU.1), and *CBFB* (all involved in blood development), and *PRDM1* [66]–[69]. *PRDM1* (BLIMP1) is involved in limb development [35], [70] and is also important for germ cell determination in mice [71]. Recent papers suggest that the regenerating blastema may acquire a germline-like state [72], [73].

Many TFs known to be involved in WNT signaling are upregulated during the middle of the time course (3 d–14 d) (*ZIC2*, *ZIC5*, *MSX2*, *SALL1*, *SALL4*, *GLI3*, *NFATC1*, *SOX4*) [74]–[79]. *WNT5A* is also upregulated during this time, and *WNT5A* has been shown to be required for the outgrowth of the limb and other structures [80]. WNT signaling has been shown to be necessary for limb and fin regeneration in zebrafish and *Xenopus*, and promotes regeneration under typically non-regenerative conditions [81]. Beta-catenin signaling has also been shown to be required for apical ectodermal ridge (AER) maintenance and for proper expression of patterning genes in the mouse [82]. Our results indicate that it is likely that WNT signaling is playing a crucial role in axolotl limb regeneration.

Keratins and collagens are upregulated during the last two time points of the time course (21 to 28 days). Keratins and collagens are also found to be enriched in regenerating *Xenopus* hind limbs when compared to non-regenerating *Xenopus* hind limbs expressing the BMP inhibitor noggin [12]. Keratins and collagens are also upregulated in the blastema of newt species [83], [84]. In addition, a gene involved in regulating size control in the hippo pathway (*MST1*), is upregulated [85]. Size regulation of the limb is probably a crucial characteristic of this phase.

### Relationship between pluripotent cells and blastema cells

Patterns of gene expression in the blastema are more similar to adult stem cells than to ES and iPS cells, in agreement with prior results investigating expression levels of embryonic stem cell genes during limb regeneration [50], [51]. This is also consistent with a recent study suggesting that the blastema is composed of a variety of progenitor cell types rather than a homogenous collection of pluripotent cells [4]. Finally, some genes highly expressed in ES/iPS cells are blastema-enriched. These genes, though, are also expressed in other cell types and are not necessarily specific to the pluripotent state. Genes in common between ES/iPS cells and blastemas, such as *KLF4* and c-MYC, are likely to be important for opening chromatin to facilitate reprogramming, but may not have specific functions related to establishing the ES cell state, as neural stem cells can be reprogrammed to the iPS state with OCT4 alone [86]. Thus the blastema bears some similarities to ES and iPS cells, but lacks the hallmarks of pluripotency (*POU5F1*, *NANOG*, *SOX2*). The observed expression patterns are more consistent with the blastema being composed of multiple adult progenitor cells, and of other mesenchymal cells [4], [10], [50], [51], [87], [88].

### Conclusions

In summary, the genes identified in this study as blastema-enriched, and active during different phases of blastema progression greatly expand the list of potential regulators of the regeneration process, providing clues as to how the axolotl regenerates its limbs and, by extension, how to potentially improve the regenerative response in mammals. To elicit a more effective regenerative response in mammals, it will be necessary to activate endogenous and exogenous genes in an appropriate, time- and pattern-controlled manner. It is feasible, however, that activation of appropriate networks would engage latent mechanisms that might simplify portions of this process. Although enhancing limb or tissue regeneration in mammals may be complicated by salamander-specific innovations [10], even partial regeneration of limb tissue or enhanced regeneration of tissues and organs has significant clinical implications.

The fact that we identify many genes previously known to be involved in the regeneration process validates our comparative RNA-seq analysis methods as does the close concordance of expression patterns between our RNA-seq data and our qPCR results. Our methods draw strength from aligning reads directly to axolotl transcript contigs while performing differential expression analysis with respect to the better characterized human gene set. This strategy will likely be beneficial in studies of transcriptomes from other non-model organisms. Although we are unable to identify the functional roles of axolotl-specific genes that play a part in regeneration with this strategy, our analyses provide lists of axolotl-specific contigs that should be studied in more depth experimentally.

We provide the data from this paper as well as other axolotl-related “omics” information at www.axolomics.org. Information available includes all the data from this manuscript including normalized gene expression measures, differential expression information, GO enrichment files, the sequencing reads, alignments, and the axolotl transcriptome assembly. In addition, several published datasets have been uploaded.

## Materials and Methods

### Axolotl husbandry, surgery, and RNA preparation

All surgical procedures and animal care were carried out in accordance with the Association for Assessment and Accreditation of Laboratory Animal Care (AAALAC) guidelines at the University of Wisconsin-Madison. We established an axolotl colony (A*mbystoma mexicanum*) from seven axolotls obtained from Dr. Gerald Eagleson (Loras College, Dubuque, IA). The animals were housed in 40% Holtfreter's salts, kept at 16–18.9°C with a pH range of 7.0–7.4. For all surgical procedures, the animals were anesthetized with 0.5–1 g/L Tricaine (MS-222, Sigma) until they were unresponsive to a tail pinch stimulus.

We amputated juvenile axolotl right forelimbs at the mid-stylopod level. For the RNA-seq experiment, the animals were 4.5–8 cm in length. For the qPCR validation experiments, the animals were 7–10 cm in length. Tissue was harvested as described in Figure 1 at 0 hours, 3 hours, 6 hours, 12 hours, 1 day, 3 days, 5 days, 7 days, 10 days, 14 days, 21 days, and 28 days. Note that for the early time points, some tissue was harvested proximal to the original amputation plane owing to the small amount of regenerative tissue distal to the amputation plane at the time of harvesting. In all cases the harvested tissues were stored in RNAlater (Qiagen, Valencia, CA) at 4°C. In all experiments, samples are placed in denaturing lysis buffer provided in the RNA isolation kit from the manufacturer and homogenized by passing through a sterile 20-gauge needle attached to a sterile plastic syringe at least 5–10 times until a homogeneous lysate is achieved. For the RNA-seq experiments, the RNA was purified using the RNeasy purification kit from Qiagen. For the qPCR experiments total RNA was isolated with the *mir*Vana miRNA isolation kit (Life Technologies) according to the manufacturer's protocol.

### Embryonic Stem (ES), Induced Pluripotent Stem (iPS), and Foreskin (FS) human RNA preparation

We cultured human FS cells (newborn foreskin fibroblasts (CRL-2097; American Type Culture Collection (ATCC)) according to ATCC recommendations. We maintained cells in 10% (v/v) FBS (Hyclone Laboratories), 1 mM L-glutamine (Invitrogen), 0.1 mM β-mercaptoethanol (Sigma-Aldrich) and 0.1 mM nonessential amino acids in DMEM (both from Invitrogen). We passaged cells at roughly 70% confluency at a 1∶3 splitting ratio, using Tryp-LE (Invitrogen). ES cells were H1 ES passage 28 cells cultured in E8 [89], harvested by direct lysis on plate with RLT lysis buffer. RLT lysis buffer is a component of the Qiagen RNeasy kit. iPS cells were DF19.7 [90] passage 27 cells cultured in E8, harvested by direct lysis on plate with RLT lysis buffer. For all three cell types, total RNA was isolated using the RNeasy kit (Qiagen).

### Sequencing library preparation and Illumina sequencing

We linearly amplified axolotl polyA+ RNAs using a modified T7 amplification method [91] resulting in cDNA. Subsequent steps followed the Illumina Paired End (PE) preparation kit (PE-102-1001, Illumina, San Diego, CA). After the Illumina PE adapters were ligated, 150–250 bp DNA fragments were isolated via gel electrophoresis. Then ten cycles of polymerase chain reaction (PCR) were performed to amplify the selected fragments using the Illumina supplied PCR primers and protocol. The sample was quantitated with the Invitrogen Qubit fluorometer (Q32857). Samples were loaded on the flowcell cluster station at a concentration of 8 pM, and sequenced on the Illumina GAII. The Genome Analyzer II Paired End recipe was used. However, for the samples in this study, only single-end data were obtained. After the sequencing was complete, the data were processed by Illumina Pipeline software for quality analysis and read filtering.

For FS cells, RNA was amplified as it was for the axolotl samples. Subsequent steps followed the Illumina Single Read (SR) preparation kit (Illumina, San Diego, CA). After the Illumina SR adapters were ligated and a NotI digestion performed for directionality, 200–300 bp DNA fragments were isolated via gel electrophoresis. Then 10 cycles of polymerase chain reaction (PCR) were performed to amplify the selected fragments using the Illumina supplied PCR primers and protocol. The sample was quantitated with the Invitrogen Qubit fluorometer (Q32857). Samples were loaded on the flowcell cluster station at a concentration of 8 pM, and sequenced on the Illumina GAII. The Genome Analyzer II SR recipe was used. After the sequencing was complete, the data were processed by Illumina Pipeline software for quality analysis and read filtering.

For ES and iPS cells, we prepared samples for sequencing using the Illumina TruSeq RNA Sample Preparation Kit v2 (RS-122-2001). The samples were quantitated with Life Technologies Qubit fluorometer (Q32857). Samples were pooled six per lane and loaded on the Illumina cbot at a final concentration of 6 pM, and sequenced on the Illumina HiSeq. The HiSeq 2000 SR multiplex recipe was used. After the sequencing was complete, the data were processed by Illumina Pipeline software for quality analysis and read filtering.

### Illumina axolotl read mapping, transcript expression determination

For axolotl transcript quantification and differential analyses, we used an axolotl contig set assembled with MIRA [92] from a combination of Sanger and 454 EST sequences. Details of the assembly process are provided in Text S1. For improved read mapping, ambiguous characters in the contigs (e.g., “N”) were replaced with one of the standard bases uniformly at random. The Illumina GA (Genome Analyzer) Pipeline v.1.4 software system was used to produce the set of sequencing reads. RSEM v1.1.6 [19], an RNA-seq quantification tool that does not require a reference genome, was used to estimate the relative abundances and expected read counts for the contigs. The default options for RSEM were used except where we specified the –no-polyA option for rsem-prepare-reference (as is appropriate for *de novo* transcriptome assemblies) and the –phred64-quals option for rsem-calculate-expression (to indicate the correct quality score encoding of the read data). By default, RSEM uses the Bowtie aligner [93] to map the reads against the contigs and we had Bowtie v0.12.1 installed for this purpose. Contigs mapping to rRNA transcripts (as determined through BLAST analyses described below) were removed and abundances were renormalized.

Because the axolotl transcriptome is largely uncharacterized, we analyzed the dynamics and functions of transcripts in the regenerating limb with respect to the human gene set. To this end, we first used BLAST (NCBI BLAST v2.2.18) to align the contigs against human RefSeq (dated 12-07-2009) [94] RNA (via BLASTN) and protein sequences (via BLASTX), taking the best BLAST hit with e-value less than 10^−5^ as the most closely related human homolog for each contig. When there was a tie for the best BLAST hit, the hit listed first was used arbitrarily. The expected read counts for contigs mapping to the same homologous human transcript were summed to give abundances. Read counts for human transcripts belonging to the same gene were summed to give human gene-level abundances. Abundances in terms of TPM were calculated by normalizing the read counts by the sums of the effective lengths (contig length – read length) of the axolotl contigs mapping to each gene and subsequently normalizing these values so that they summed to one million.

Expected read counts were used as input to differential expression analysis by EdgeR (version 3.0.0, R version 2.1.5) [25]. Because we only had one biological replicate per time point, we compared pairs of samples from consecutive time points to obtain estimates of biological variation for DE analyses. EdgeR was used to estimate the common dispersion factor for each pair of samples from consecutive time points and the median of these values was used as the common dispersion factor for our DE analyses. For our comparative method, which uses counts at the human gene level, the common dispersion was 0.05, whereas for the contig-level approach, this value was 0.11. With the appropriate common dispersion factor, EdgeR was used to predict the set of DE genes or contigs at each time point with respect to the zero hour control sample. Unless otherwise specified, genes were determined to be DE by application of one criterion: a Benjamini-Hochberg [95] adjusted p-value of less than 0.05.

### Data analysis for ES, iPS and FS cell samples

For the FS samples, RNA-seq results were calculated using Illumina's Casava 1.7 pipeline and RSEM version 1.1.7, aligned to the hg18 genome build [19]. For the ES and iPS samples, Casava 1.8.2 and RSEM 1.1.21 were used, aligned to the hg19 genome build. By default, RSEM uses the bowtie short-read aligner. For FS, ES, and iPS cells, RSEM was run with Bowtie pass-through parameters m = 200, n = 2, and seed-length = 28, using Bowtie version 0.12.7 [93].

### Analysis of the completeness of the axolotl assembly

From each of the juvenile time course samples (average total number of reads ∼17.5 million), we extracted all very high quality reads (average phred score > = 38) and partitioned them into two sets: those that had an alignment against the axolotl assembly (AL = aligned) and those that did not (UN = unaligned). Only the highest quality reads were selected to reduce bias in later steps because lower quality reads are less likely to be alignable. This step resulted in ∼5 million reads per sample on average (∼2.8 million aligned and ∼2.2 million unaligned on average). We ran BLASTX with these reads against the human RefSeq protein set with an e-value cutoff of 1e-5 and a single top hit for each read was reported.

For each read, we consider the following possible events: it has an alignment to the axolotl assembly (AL); it does not have an alignment to the axolotl assembly (UN); it is truly derived from an axolotl transcript (A); it is not derived from an axolotl transcript (NA); it has a BLASTX hit (B); and it has average phred score > = 38 (HQ). For the BLASTX analysis of the UN reads, we have
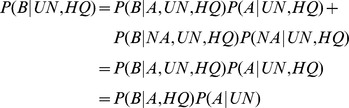
where in the second line we assume that NA reads will not have any BLASTX hits, as these are presumably RNA-seq protocol artifacts, and in the third line, we assume that the quality of a read is independent of whether it is derived from axolotl and that having a BLASTX hit is independent of whether the read is aligned (AL), given that it is truly from axolotl. Then, considering the BLASTX analysis of the AL reads, we have
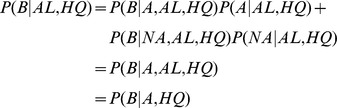
where in the second line we assume that all AL reads are from axolotl and in the third line we again assume that having a BLASTX hit is independent of whether the read as aligned (AL), given that it is truly from axolotl. Putting these two equations together, we have
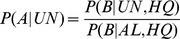
and thus we can estimate the fraction of UN reads that are truly from axolotl from the fractions of the UN HQ and AL HQ reads that have BLASTX hits. To estimate the fraction of reads that are from axolotl but were unalignable, we simply compute 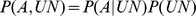. In the case of false positive BLASTX hits from NA reads, we will overestimate 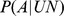 because such hits will cause our estimate of 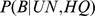 to be higher. Therefore our estimates of the completeness of the assembly are conservative.

### GO analysis and clustering

We used DAVID (version 6.7) to perform Gene Ontology (GO) analysis [49]. The statistically significant (FDR<0.05) upregulated genes compared to the zero hour control were used as input to DAVID. Each time point's upregulated genes were submitted to DAVID functional analysis chart GO analysis, with the background set being the set of human genes to which axolotl contigs had significant (e-value<1e-5) best BLAST hits and had at least one matching read in our RNA-seq data. (The background set is available at axolomics.org, as are all of the DAVID result files.) For DAVID analysis, the following settings were used: categories selected are GOTERM_BP_5, GOTERM_MF_5; threshold options are Counts = 2, EASE = 0.1). The GO heat map (Figure 6) was constructed by creating a table of the DAVID FDR scores for each GO term with an FDR<0.01 at any time point. The negative log base 10 of these FDR values was used in the heat map, and these values are shown in Table S4. MeV 4.8.1 (http://www.tm4.org/mev/) was used to cluster the GO terms (hierarchical clustering with distance measure = Pearson Correlation, linkage = average).

We performed sample and gene clustering using MeV 4.8.1. Hierarchical cluster analyses were carried out with Pearson uncentered correlation (Figure 3, Figure 4) or Pearson correlation (Figure 6) as the distance measurement with average linkage. Pearson uncentered correlation was used for Figures 3 and 4 because log fold changes are expected to be centered around zero (no fold change), whereas Pearson correlation was used for Figure 6 because −log(FDR) values are all non-negative. Clusters and heat maps were visualized via MeV 4.8.1.

To assess the uncertainty in hierarchical cluster analysis over samples, we applied bootstrap resampling (10,000 iterations) via the R package Pvclust [26]. The uncentered Pearson correlation is used as the distance metric with average linkage. Pvclust provides the Bootstrap Probability (BP) value from the ordinary bootstrap resampling [96] and the Approximately Unbiased (AU) probability value from multiscale bootstrap resampling [26], [97]. The ordinary bootstrap resampling method has been shown to be biased especially when genes are correlated. The multiscale bootstrap resampling method was introduced to develop an approximately unbiased test, and therefore it provides better estimations of the probability values [26], [97]. Let N denote the original number of genes. In multiscale resampling, instead of resampling N genes in each of 10,000 bootstraps, we resampled the genes with 10 different data sizes (as is the default setting of the package). The resampled data sizes vary from 0.6*N to 1.4*N. The numbers above each edge show the probability of nodes below that edge occurring as a cluster in resampled trees, via ordinary bootstrap resampling (BP, green) or multiscale bootstrap resampling (AU, red).

### Transcription factor list

We generated a list of human TFs by querying genes for GO terms that match: “RNA polymerase I transcription factor activity”, “RNA polymerase II transcription factor activity”, “RNA polymerase II transcription factor activity, enhancer binding”, “RNA polymerase III transcription factor activity”, “transcription factor activity”, “transcription activator activity”. We also added genes with “transcription factor” in their refseq gene functional description, giving a total of 2260 TFs. The TF file, DE gene lists, and all the RNA-seq data are available at axolomics.org.

### RNA isolation for reverse transcription and real-time quantitative PCR (qPCR)

We isolated total RNA with the *mir*Vana miRNA isolation kit (Life Technologies) according to the manufacturer's protocol. Total RNA (an input range between 88 to 808 ng/µl) was treated with DNaseI (Life Technologies) for 10 min at 37°C followed by heat inactivation for 5 min at 65°C. DNaseI-treated total RNA was combined with (oligo-dT)_15_ primer and denatured by heating to 70°C for 5 min and chilling to −4°C for 5 minutes. The denatured (oligo-dT)_15_ primer and RNA was reverse transcribed by adding the ImPromII reverse transcription system (Promega) to generate cDNA using the following program: 5 min at 25°C, 60 min at 42°C, and 15 min at 70°C to inactivate the reverse transcriptase. Total cDNA was diluted five-fold and used at 1 µl per 10 µl of qPCR reaction with TaqMan Universal PCR Master Mix (Life Technologies). Axolotl gene specific qPCR assays were designed using PrimerQuest for PrimeTime qPCR assays (Integrative DNA Technologies). Sequences for gene specific oligos are listed below. The qPCR reactions were performed with ViiA 7 Real-Time PCR System (Life Technologies) under the following cycle conditions: 2 min at 50°C, 10 min at 95°C followed by 40 cycles of 15 sec at 95°C, 1 min at 60°C. To quantify the relative expression level of a particular gene, three independent qPCR reactions (technical replicates) were performed on biological triplicate samples for each time point analyzed, unless otherwise stated. All data points were normalized to GAPDH and relative to samples collected at the 0 hour time point using the comparative C_T_ (ΔΔCT) method. Primer sequences for qPCR are in Table S5.

### qPCR data analysis

For comparing RNA-seq data to qPCR data, the data were analyzed as follows: For RNA-seq data, the log_2_ ratio = log_2_ (TPM at time point x+1)/(TPM at time zero+1). One was added to TPMs to avoid divide by zero errors, log(0) errors, and to avoid obtaining a high ratio when both TPMs are low. For qPCR, ΔCT is defined as the difference in the cycle threshold (CT) between the gene of interest and the GAPDH control. For qPCR data, the log2 ratio = log_2_(ΔCT at time point x)/(ΔCT at time zero). Any ΔCT ratios of 0 were changed to 0.1 before log transformation to avoid error. For genes with undetectable levels from samples collected at 0 hour, CT values were assigned at 40 to avoid error.

Figure 2 contains plots of the log2 ratios of RNA-seq and qPCR for four genes. The plots for the additional 15 genes validated by qPCR are shown in Figure S2. Upon analysis of this data, we noticed that for genes with a late peak (limb genes such as HOXD10), the qPCR peak is often delayed compared to the RNA-seq peak. The animals used for the qPCR experiment were slightly larger than the animals used for the RNA-seq experiment (7–10 cm for qPCR, vs. 4.5–8 cm for RNA-seq). It is well known that larger animals regenerate more slowly. Because of this lag in the later qPCR time points, when we calculate the Pearson correlation, we remove the 3 d time point from the qPCR data and then “shift left” the remaining qPCR time points. Thus we compare the 3 hr–21 d RNA-seq samples to the 3 hr–1 d plus 5 d–28 d qPCR samples. This comparison was used for the Pearson correlation calculation.

## Supporting Information

Figure S1 **Number of genes with a Transcripts Per Million (TPM) measurement greater than one.**
(TIF)

Figure S2 **Expression patterns of RNA-seq and qPCR along the time course.** Plots of the log_2_ ratios of each time point relative to the zero hour control for both the RNA-seq (blue) and the qPCR data (red) for 15 additional genes. The R^2^ Pearson correlation across the time course is also shown for each gene. The general trends between the two methods match well across the time course for the majority of genes (average Pearson correlation for all 19 genes = 0.741). See Materials and Methods for an explanation of determining the ratios and of the Pearson calculation. See also Figure 2 for four other RNA-seq vs qPCR plots.
(EPS)

Figure S3 **Heat map of limb genes in juvenile blastemas ratioed to the zero hour juvenile blastema control.** The list of limb genes was gathered from the literature. Many limb genes peak at 10 to 14 days.
(TIF)

Figure S4 **Distributions of the lengths of the longest ORF found within Differentially Expressed (DE) contigs.** The length is represented as a fraction of the contig length. Separate distributions are provided for DE contigs with and without a significant BLAST hit to a human gene. Also shown are the distributions for the DE contigs with randomly shuffled sequences, which lack any true coding potential.
(TIF)

Figure S5 **The number of Differentially Expressed (DE) genes at each time point (FDR<0.05).**
(TIF)

Figure S6 **Heat map of pairwise Pearson correlation coefficients (R^2^) between samples in the time course.** Note that the early (0 hr through 12 hr) pairwise comparisons have increased Pearson correlations, as do the 3 d through 21 d pairwise comparisons, and the 21 d and 28 d comparison.
(TIF)

Table S1 **Number of reads and mapping rates to axolotl contigs and human genes for each time point sample.**
(XLSX)

Table S2 **RNA-seq and qPCR log_2_ ratios relative to the time zero controls for all 19 genes.** See Materials and Methods for details.
(XLSX)

Table S3 **Pairwise Pearson correlation coefficients from the heat map of Figure S6.**
(DOCX)

Table S4 **The −log_10_(GO term FDR) numbers for the heat map of** **Figure 6** **.**
(XLSX)

Table S5 **Primer sequences used in qPCR experiments.**
(XLSX)

Text S1 **Supplemental experimental procedures.**
(DOCX)
