# Supplementary figures and images for: Comparative RNA-seq Analysis in the Unsequenced Axolotl: The Oncogene Burst Highlights Early Gene Expression in the Blastema
Source: PLoS Comput Biol. 2013 Mar 7;9(3):e1002936. doi: 10.1371/journal.pcbi.1002936 (PMC3591270; doi:10.1371/journal.pcbi.1002936)

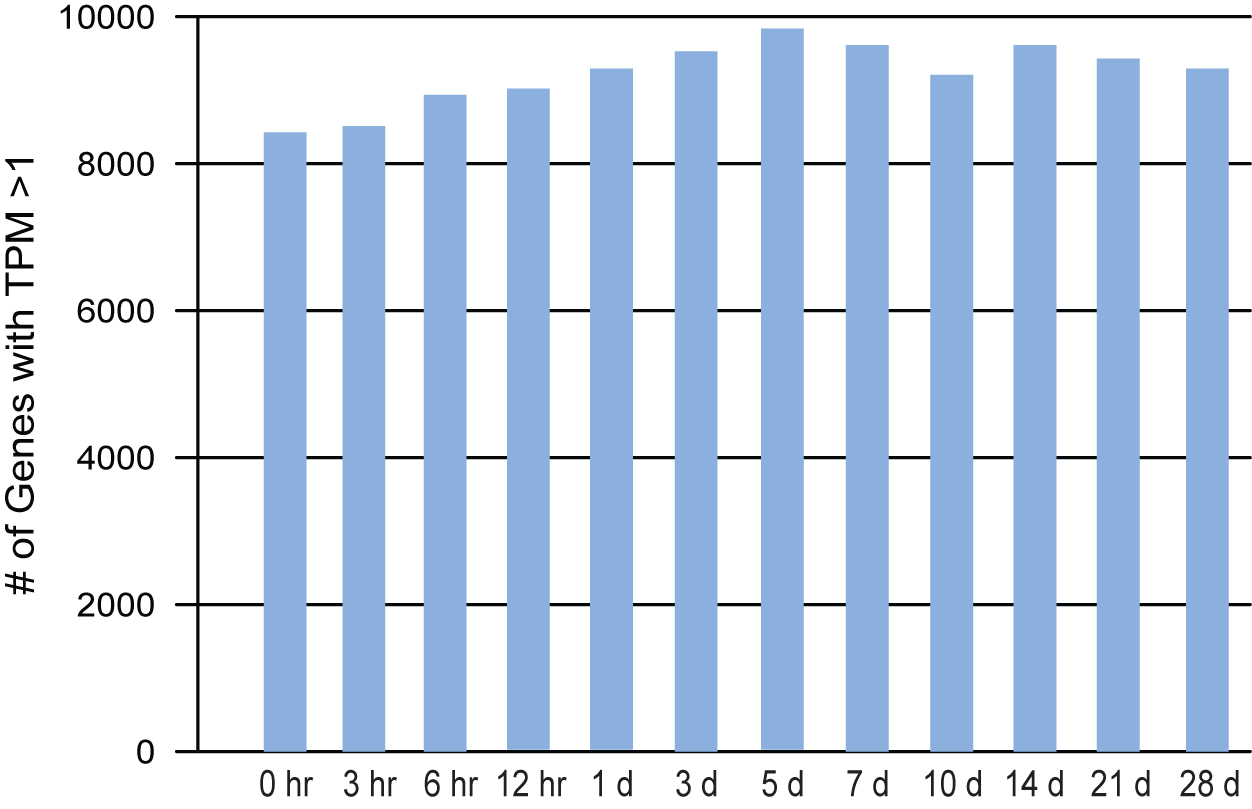

Supplement: Figure S1 — Number of genes with a Transcripts Per Million (TPM) measurement greater than one. (TIF) [file pcbi.1002936.s001.tif]

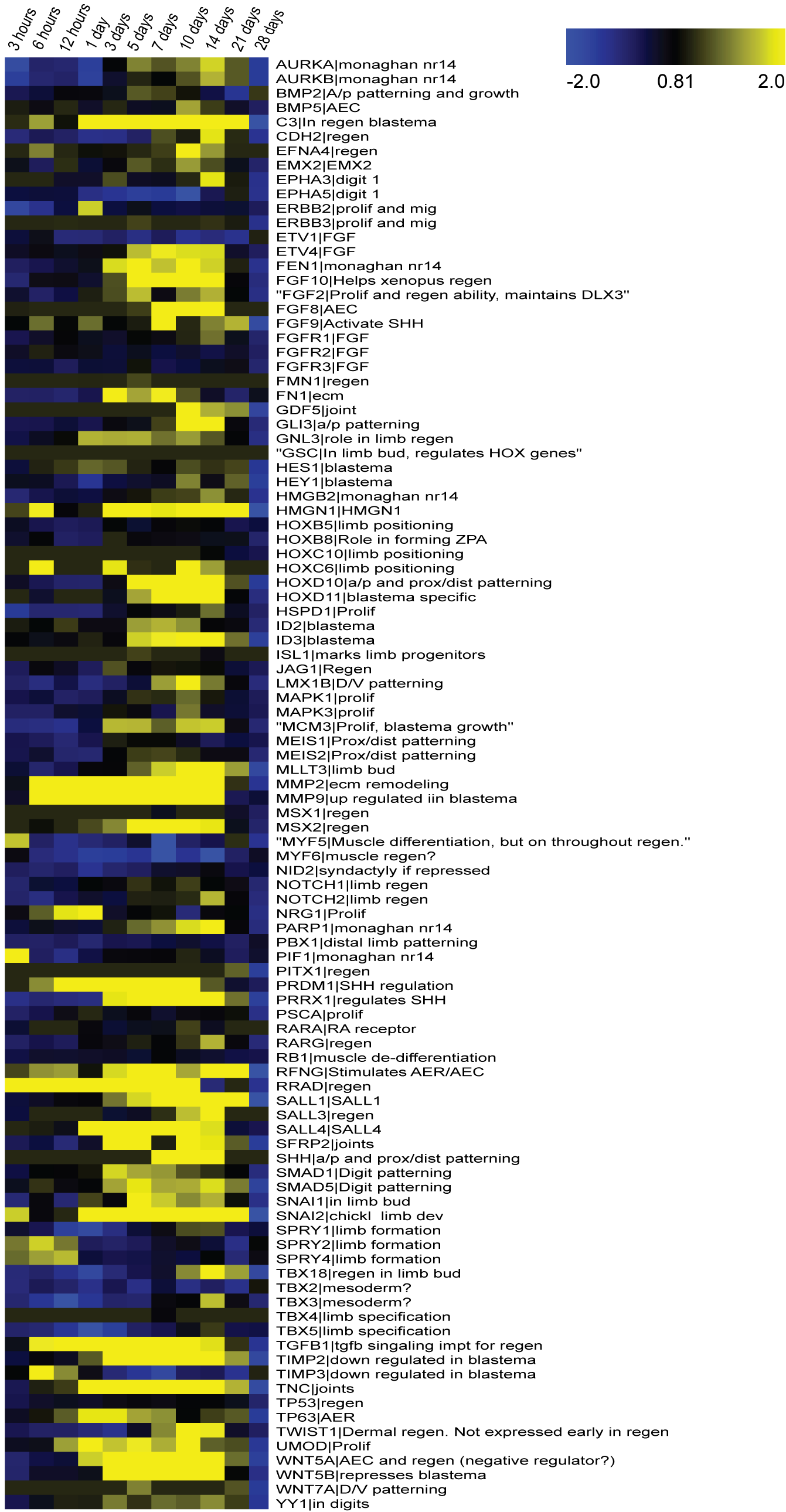

Supplement: Figure S3 — Heat map of limb genes in juvenile blastemas ratioed to the zero hour juvenile blastema control. The list of limb genes was gathered from the literature. Many limb genes peak at 10 to 14 days. (TIF) [file pcbi.1002936.s003.tif]

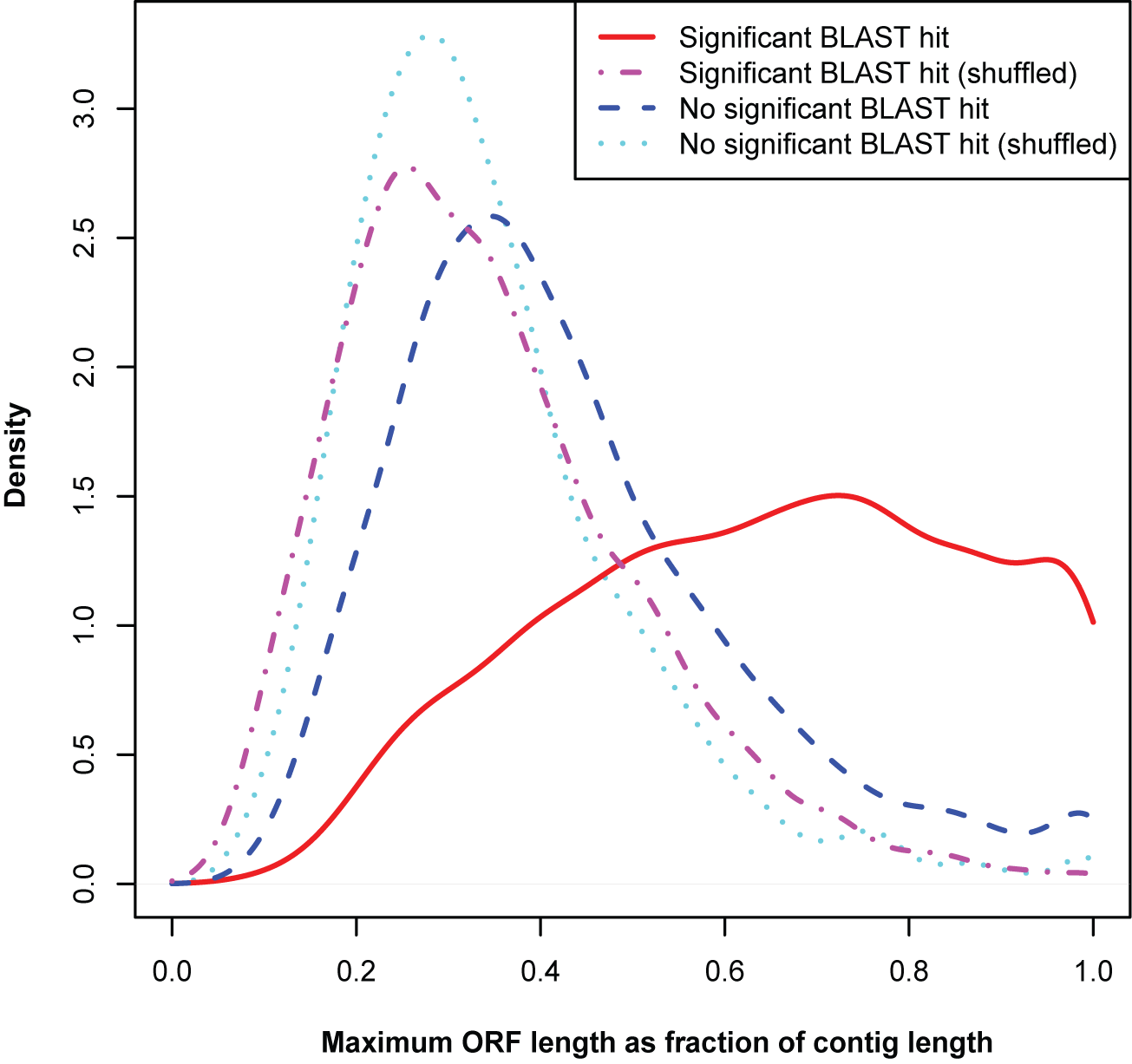

Supplement: Figure S4 — Distributions of the lengths of the longest ORF found within Differentially Expressed (DE) contigs. The length is represented as a fraction of the contig length. Separate distributions are provided for DE contigs with and without a significant BLAST hit to a human gene. Also shown are the distributions for the DE contigs with randomly shuffled sequences, which lack any true coding potential. (TIF) [file pcbi.1002936.s004.tif]

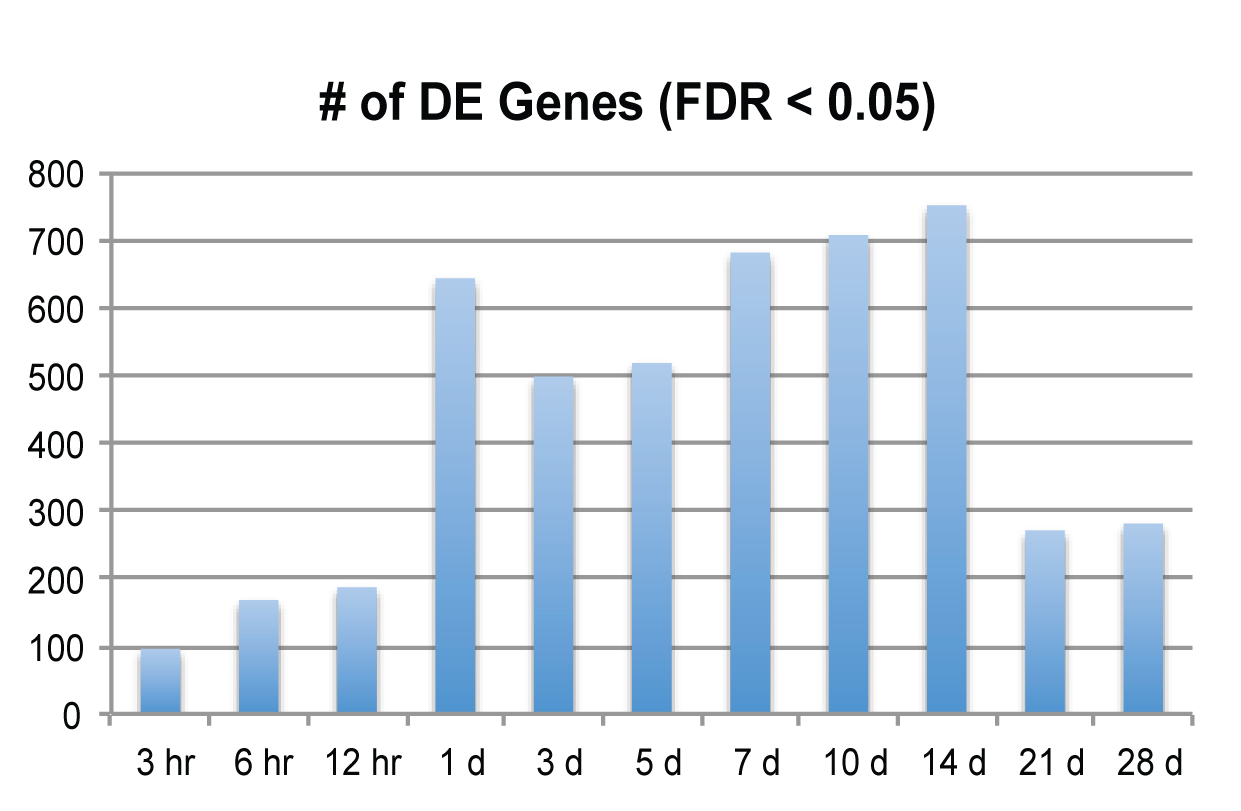

Supplement: Figure S5 — The number of Differentially Expressed (DE) genes at each time point (FDR<0.05). (TIF) [file pcbi.1002936.s005.tif]

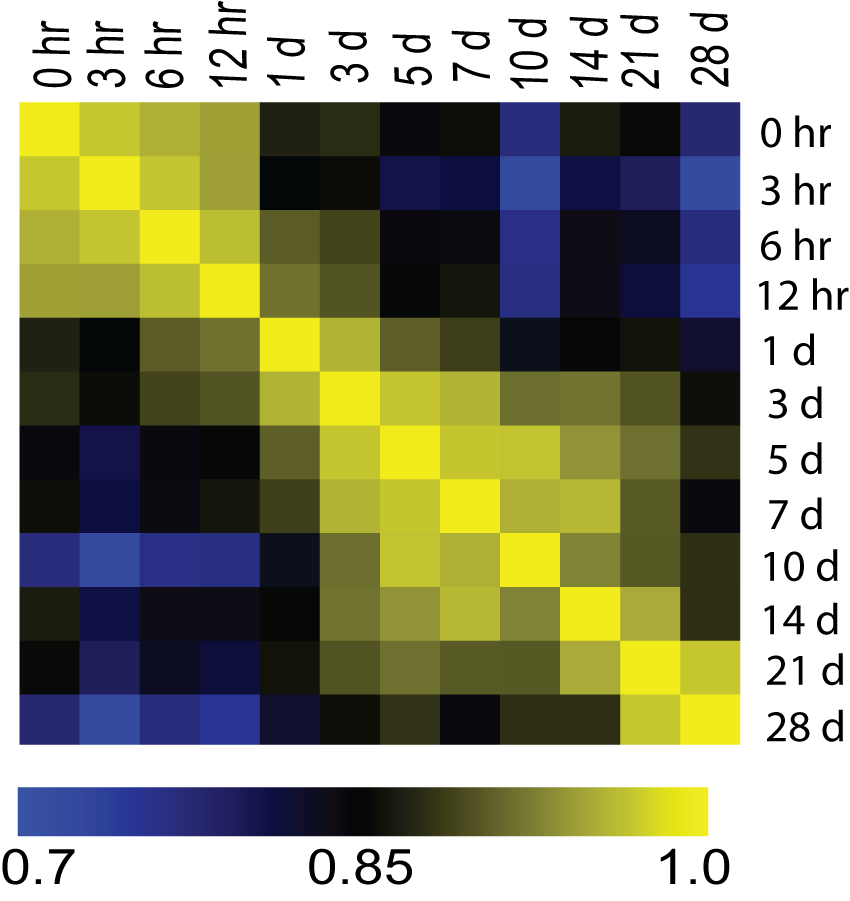

Supplement: Figure S6 — Heat map of pairwise Pearson correlation coefficients (R2) between samples in the time course. Note that the early (0 hr through 12 hr) pairwise comparisons have increased Pearson correlations, as do the 3 d through 21 d pairwise comparisons, and the 21 d and 28 d comparison. (TIF) [file pcbi.1002936.s006.tif]
