# Supplementary material for: Comparative RNA-seq Analysis in the Unsequenced Axolotl: The Oncogene Burst Highlights Early Gene Expression in the Blastema
Source: PLoS Comput Biol. 2013 Mar 7;9(3):e1002936. doi: 10.1371/journal.pcbi.1002936 (PMC3591270; doi:10.1371/journal.pcbi.1002936)
Supplement: Table S3 — Pairwise Pearson correlation coefficients from the heat map of Figure S6. (DOCX) [file pcbi.1002936.s009.docx]

**Table S3.**

|  | 0 hr | 3 hr | 6 hr | 12 hr | 1 d | 3 d | 5 d | 7 d | 10 d | 14 d | 21 d | 28 d |
| --- | --- | --- | --- | --- | --- | --- | --- | --- | --- | --- | --- | --- |
| 0 hr | 1 | 0.9679 | 0.954 | 0.9444 | 0.8684 | 0.8758 | 0.8427 | 0.8563 | 0.769 | 0.8648 | 0.8538 | 0.7763 |
| 3 hr | 0.9679 | 1 | 0.9664 | 0.9436 | 0.85 | 0.8548 | 0.8052 | 0.8103 | 0.7203 | 0.8093 | 0.7936 | 0.7182 |
| 6 hr | 0.954 | 0.9664 | 1 | 0.9618 | 0.9034 | 0.8899 | 0.8422 | 0.8403 | 0.7626 | 0.8361 | 0.8286 | 0.7688 |
| 12 hr | 0.9444 | 0.9436 | 0.9618 | 1 | 0.9165 | 0.8999 | 0.8518 | 0.8616 | 0.7654 | 0.8378 | 0.8136 | 0.7526 |
| 1 d | 0.8684 | 0.85 | 0.9034 | 0.9165 | 1 | 0.9548 | 0.9049 | 0.8869 | 0.8325 | 0.8521 | 0.8577 | 0.8209 |
| 3 d | 0.8758 | 0.8548 | 0.8899 | 0.8999 | 0.9548 | 1 | 0.9665 | 0.9554 | 0.9145 | 0.9181 | 0.8985 | 0.8567 |
| 5 d | 0.8427 | 0.8052 | 0.8422 | 0.8518 | 0.9049 | 0.9665 | 1 | 0.9674 | 0.9657 | 0.936 | 0.9156 | 0.8798 |
| 7 d | 0.8563 | 0.8103 | 0.8403 | 0.8616 | 0.8869 | 0.9554 | 0.9674 | 1 | 0.9539 | 0.9578 | 0.9028 | 0.8413 |
| 10 d | 0.769 | 0.7203 | 0.7626 | 0.7654 | 0.8325 | 0.9145 | 0.9657 | 0.9539 | 1 | 0.9265 | 0.9108 | 0.8773 |
| 14 d | 0.8648 | 0.8093 | 0.8361 | 0.8378 | 0.8521 | 0.9181 | 0.936 | 0.9578 | 0.9265 | 1 | 0.9502 | 0.8773 |
| 21 d | 0.8535 | 0.7936 | 0.8286 | 0.8136 | 0.8577 | 0.8985 | 0.9156 | 0.9028 | 0.9018 | 0.9502 | 1 | 0.9681 |
| 28 d | 0.7763 | 0.7182 | 0.7688 | 0.7256 | 0.8209 | 0.8567 | 0.8798 | 0.8413 | 0.8778 | 0.8773 | 0.9681 | 1 |
