# Supplementary material for: Comparative RNA-seq Analysis in the Unsequenced Axolotl: The Oncogene Burst Highlights Early Gene Expression in the Blastema
Source: PLoS Comput Biol. 2013 Mar 7;9(3):e1002936. doi: 10.1371/journal.pcbi.1002936 (PMC3591270; doi:10.1371/journal.pcbi.1002936)
Supplement: Text S1 — Supplemental experimental procedures. (DOCX) [file pcbi.1002936.s012.docx]

**Comparative RNA-seq Analysis in the Unsequenced Axolotl: The Oncogene Burst Highlights Early Gene Expression in the Blastema**

Ron Stewart^1^, Cynthia Alexander Rascón^2^, Shulan Tian^1^, Jeff Nie^1^, Chris Barry^1^, Li-Fang Chu^1^, Hamisha Ardalani^8^, Ryan J. Wagner^1^, Mitchell D. Probasco^1^, Jennifer M. Bolin^1^, Ning Leng^7^, Srikumar Sengupta^1^, Michael Volkmer^3^, Bianca Habermann^3^, Elly M. Tanaka^2^, James A. Thomson^1,4,5^, Colin N. Dewey^6^

^1^Regenerative Biology, Morgridge Institute for Research, Madison, WI 53707, USA;

^2^Technical University Dresden, DFG Center for Regenerative Therapies Dresden, Fetscherstrasse 105 01039 Dresden, Germany;

^3^Max-Planck-Institute for Biology of Ageing, Cologne, Germany;

^4^Department of Cell & Regenerative Biology, School of Medicine and Public Health, University of Wisconsin, Madison, WI 53707, USA;

^5^Department of Molecular, Cellular, & Developmental Biology, University of California Santa Barbara, Santa Barbara, CA 93106, USA;

**^6^**Department of Biostatistics and Medical Informatics, University of Wisconsin, Madison, WI 53706-1510, USA.

**^7^**Department of Statistics, University of Wisconsin, Madison, WI 53706, USA;

^8^Department of Biomedical Engineering, University of Wisconsin, Madison, WI 53705, USA.

**Inventory of Supplementary Information:**

The supplementary information consists of 6 figures and 5 tables.

Figure S1

Figure S2

Figure S3

Figure S4

Figure S5

Figure S6

Table S1

Table S2

Table S3

Table S4

Table S5

**TEXT S1**

**SUPPLEMENTAL EXPERIMENTAL PROCEDURES**

**Protocol for the Assembly of Axolotl EST Sequences**

The EST reads for the assembly come from:

1) 33,152 EST Sanger trace files obtained from Elly Tanaka lab’s sequencing (ET) of a mixture of axolotl cell types including embryos at stages 12, 14, 27 and 40; limb buds, limb blastemas at 15 h, 30 h, 44 h, 60 h, 4 d, 6 d, 8 d, and 13 d after amputation; and tail blastemas at 1 d and 7 d after amputation, mature spinal cord and spinal cord cell culture.

2) 22,237 EST Sanger trace files from Randal Voss's (RV) sequencing effort.

For Sanger sequences from the Voss and Tanaka labs, all *.scf, *.esd and *.ab1 traces files were used. Suffixes were added to Sanger sequences according to St. Louis convention to indicate sequencing direction.

3) 1,208,954 EST 454 sequencing reads, with the corresponding quality scores, from ET.

4) 391 Axolotl NCBI sequences (without sequencing quality and hence taken as the assembler's default values) present in a previous ET Sanger assembly (Habermann et al., 2004).

To retrieve base calls and quality values from the sequencer trace data

for each Sanger read, PHRED (Version: 0.020425.c, [www.phrap.org/)](http://www.phrap.org/)) was used. 454 base calls and quality files were given by the sequencing provider. NCBI fasta sequences were included as if they were Sanger reads with no quality score.

All reads were masked using CrossMatch version 1.090518 (<http://www.phrap.org/)> for vector sequences (revEXPRESS, pCMV-SporT 6, pCr2.1-Topo, pCR4-TOPO, pExpress-1.fasta and cloning vector pTriplEx) contained in the UniVec database from NCBI (<ftp://ftp.ncbi.nih.gov/pub/UniVec/> March 2010), and vector sequences used

in the ET and RV libraries.

MIRA 3.0.0 assembler (Bastien Chevreux,

<http://www.chevreux.org/projects_mira.html)> was used to perform the hybrid

de novo assembly of the masked Sanger (ET and RV) sequences, together with

the ET 454 sequences. (job=denovo,est,normal,sanger,454 -notraceinfo SANGER_SETTINGS-LR:rns=stlouis -OUT:sssip=1 454_SETTINGS -CL:cpat=on -OUT:sssip=1).

The resulting assembly consisted of 113,925 contigs with

an N50 contig size of 650 (median=500, mean=623, max=7943).

Num. reads assembled: 1,048,993

Num. singlets: 43,725

Number of contigs: 107,987 **(made up from >=2 reads)**

Max coverage=377, Average consensus quality=35 (note that default base quality of reads that have no quality read from file is 10).

*5,938 singlets at some point were part of a larger alignment in the first passes or part of potentially 'important' decisions within the assembly according to MIRA, and remained in the contigs output file.

**SUPPLEMENTAL REFERENCES**

Habermann B, Bebin A-G, Herklotz S, Volkmer M, Eckelt K, et al. (2004). An Ambystoma mexicanum EST sequencing project: analysis of 17,352 expressed sequence tags from embryonic and regenerating blastema cDNA libraries. Genome Biology. *5*, R67.
